# Persistent Reptarenavirus and Hartmanivirus Infection in Cultured Boid Cells

**DOI:** 10.1128/spectrum.01585-22

**Published:** 2022-07-07

**Authors:** Annika Lintala, Leonora Szirovicza, Anja Kipar, Udo Hetzel, Jussi Hepojoki

**Affiliations:** a University of Helsinki, Faculty of Medicine, Medicum, Department of Virology, Helsinki, Finland; b University of Zürich, Vetsuisse Faculty, Institute of Veterinary Pathology, Zürich, Switzerland; c University of Helsinki, Faculty of Veterinary Medicine, Department of Basic Veterinary Sciences, Helsinki, Finland; Erasmus MC

**Keywords:** arenavirus, cell culture, coinfection, persistence

## Abstract

Mammarenaviruses establish a persistent infection in their rodent and bat hosts, and the evidence suggests that reptarenaviruses and hartmaniviruses found in captive snakes act similarly. In snakes, reptarenaviruses cause boid inclusion body disease (BIBD), which is often associated with secondary infections. Snakes with BIBD usually carry more than a single pair of reptarenavirus S and L segments and occasionally demonstrate hartmanivirus coinfection. Here, we reported the generation of cell lines persistently infected with a single or two reptarenavirus(es) and a cell line with persistent reptarenavirus-hartmanivirus coinfection. By RT-PCR we demonstrated that the amount of viral RNA within the persistently infected cells remains at levels similar to those observed following initial infection. Using antibodies against the glycoproteins (GPs) and nucleoprotein (NP) of reptarenaviruses, we studied the levels of viral protein in cells passaged 10 times after the original inoculation and observed that the expression of GPs declines dramatically during persistent infection, unlike the expression of NP. Immunofluorescence (IF) staining served to demonstrate differences in the distribution of NP within the persistently infected compared to freshly infected cells. IF staining of cells inoculated with the viruses secreted from the persistently infected cell lines produced similar NP staining compared to cells infected with a traditionally passaged virus, suggesting that the altered NP expression pattern of persistently infected cells does not relate to changes in the virus. The cell cultures described herein can serve as tools for studying the coinfection and superinfection interplay between reptarenaviruses and studying the BIBD pathogenesis mechanisms.

**IMPORTANCE** Mammarenaviruses cause a persistent infection in their natural rodent and bat hosts. Reptarenaviruses cause boid inclusion body disease (BIBD) in constrictor snakes, but it is unclear whether snakes are the natural host of these viruses. In this study, we showed that reptarenaviruses established a persistent infection in cultured Boa constrictor cells and that the persistently infected cells continued to produce infectious virus. Our results showed that persistent infection results from subsequent passaging of cells inoculated with a single reptarenavirus, two reptarenaviruses, or even when inoculating the cells with reptarenavirus and hartmanivirus (another arenavirus genus). The results further suggested that coinfection would not result in overt competition between the different reptarenaviruses, thus helping to explain the frequent reptarenavirus coinfections in snakes with BIBD. The established cell culture models of persistent infection could help to elucidate the role of coinfection and superinfection and potential immunosuppression as the pathogenic mechanisms behind BIBD.

## INTRODUCTION

Since their discovery in the 1930s, the members of the family *Arenaviridae* were associated with rodent hosts, except for the Tacaribe virus found in bats. However, the identification of arenaviruses from captive snakes in 2012 broadened the scope of potential hosts (1–4). Those discoveries led to a series of changes in arenavirus taxonomy. The first revision established two novel genera, *Mammarenavirus* and *Reptarenavirus*, to house the viruses found in rodents and bats, and those found in snakes (4). Sequencing studies of reptarenavirus isolates led us to identify Haartman Institute snake virus 1 (HISV-1) in 2015 (5), and further characterization of the isolated virus (6) led to the formation of the novel arenavirus genus *Hartmanivirus* in 2018 (7). The identification of divergent arenaviruses, Wēnlǐng frogfish arenaviruses 1 and 2 as well as salmon pescarenavirus 1, in fish in 2018/2019 (8, 9) led to the establishment of the genus *Antennavirus* in 2019 and expanded the family *Arenaviridae* to comprise four genera (10). Due to the historical perspective, arenavirology largely builds on the knowledge gathered from mammarenavirus studies, whereas the information about and studies on the other arenavirus genera lag. The single-stranded RNA genome of arenaviruses is bisegmented (S and L segment), except for antennaviruses that are trisegmented (S, M, and L) (11). For the bisegmented arenaviruses, the L segment encodes the zinc finger matrix protein (ZP) and RNA-dependent RNA polymerase (RdRp), except for hartmaniviruses that lack the ZP (6), while the S segment encodes the glycoprotein precursor (GPC) and nucleoprotein (NP) (11).

Descriptions of boid inclusion body disease (BIBD), a disease considered eventually lethal, in boid snakes go back to the 1970s (12). BIBD manifests as the formation of inclusion bodies (IBs) in various cell types of affected snakes and the disease is associated with central nervous system (CNS) signs (12). Curiously, given the 40-year history of the disease descriptions, three independent groups identified reptarenaviruses in constrictor snakes (boas and pythons) with BIBD in the 2010s (1–3). Both Hetzel et al. (1) and Stenglein et al. (3) showed that reptarenavirus infection causes IB formation and that the IBs comprise reptarenavirus NP, suggesting a causal link between the virus and BIBD. Stenglein et al. (13) experimentally reproduced BIBD through inoculation of pythons and boas with isolated reptarenavirus via cardiac venepuncture. The study reported classical BIBD, characterized by IB formation in various tissues in boas, whereas the infected pythons showed predominantly CNS signs (13). We were unable to reproduce BIBD in boas using tracheal inoculation. However, we could reproduce infection with CNS signs in pythons (14). Snakes with BIBD often carry multiple reptarenavirus S and L segments (5, 15), and they can vertically transmit some or all their segments to their offspring (16). Both naturally and experimentally reptarenavirus infected snakes mount an antibody response against the main component of the IBs, i.e., reptarenavirus NP (14, 17, 18). However, the anti-NP response appears to correlate inversely with the presence of IBs because naturally infected snakes demonstrated higher reptarenavirus-neutralizing antibody responses than the experimentally infected snakes (14, 18). Snakes are poikilotherm and their immune response is temperature-dependent (19) as is reptarenavirus growth (20), and we speculate that these factors affect the course of reptarenavirus infection and BIBD pathogenesis in snakes (14). Mammarenaviruses establish a persistent infection in their respective hosts and each mammarenavirus infects a specific or closely related rodent species, suggesting virus-host codivergence in evolution (21). Rodents with persistent mammarenavirus infection can mount a measurable antibody response against the virus. However, significant differences in the induced response may occur between virus strains (21).

We initiated this study to evaluate whether reptarenaviruses and hartmaniviruses would act similarly to mammarenaviruses in cell culture in that they would establish a persistent infection. For this study, we selected one single reptarenavirus isolate (i.e., a pair of S and L segments), one isolate with two reptarenaviruses (two S and two L segments), and one isolate containing a reptarenaviruse and a hartmanivirus. We used quantitative real-time PCR (qRT-PCR) and immunoblot to study virus replication and protein expression during passaging of the infected cells, and immunofluorescence (IF) analysis to study the expression of viral NP and the glycoproteins (GPs) in the persistently infected cell cultures.

## RESULTS

### Generation and evaluation of virus segment-specific quantitative reverse transcription PCR (qRT-PCR).

**FIG 1:**
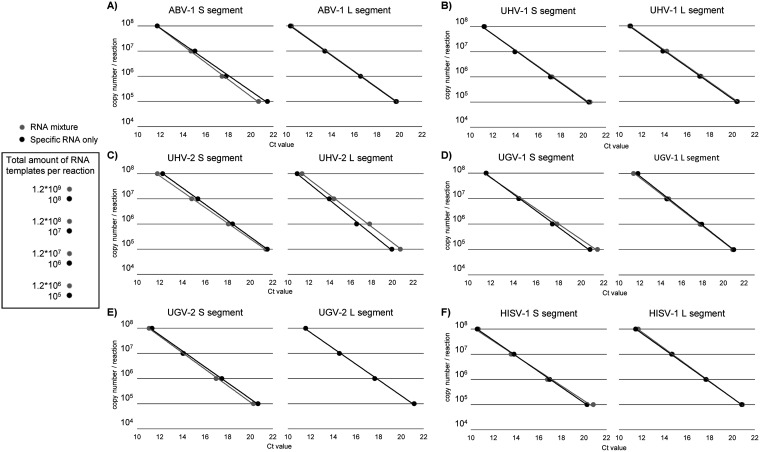
Performance and specificity of qRT-PCRs targeting S and L segments. *In vitro* transcribed synthetic RNAs served for testing the specificity of the primer and probe combinations targeting the S and L segments of ABV-1, UGV-1, UGV-2, UHV-1, UHV-2, and HISV-1. The one-step qRT-PCRs included a 10-fold dilution series ranging from 100,000 to 100,000,000 copies per reaction of the specific target alone or in mixture with the RNA targets of the other primer-probe combinations. The plots show the performance of (A) ABV-1 S and L segment, (B) UHV-1 S and L segment, (C) UHV-2 S and L segment, (D) UGV-1 S and L segment, (E) UGV-2 S and L segment, (F) HISV-1 S and L segment targeting primers and probe with (gray line) and without (black line) the synthetic RNA targets of other viruses and segments. The *y*-axis represents the copy number in reaction, and the *x*-axis shows the respective Ct values.

We designed S and L segment-specific one-step qRT-PCR assay for aurora borealis virus 1 (ABV-1), University of Giessen virus 1 and 2 (UGV-1 and -2), University of Helsinki virus 1 and 2 (UHV-1 and -2), and Haartman Institute Snake virus 1 (HISV-1). To enable the translation of Ct values to copy numbers, we used *in vitro* transcription to produce control RNAs (approximately 450 to 500 nt depending on the target) from plasmids bearing synthetic gene blocks. Fig. 1 shows the performance of each set of primers and probes (sequences provided in Table 1) with the specific template alone and in a mixture with all the other control RNAs. The presence of S and L segment templates of other reptarenaviruses did not markedly affect the amplification specificity of the primer and probe combinations. By titrating the specific control RNAs, we further observed that each set of primers and probes was able to detect less than 10 copies of the specific control RNA per reaction.

**TABLE 1:** Sequences of the primers and probes utilized in qRT-PCR

Virus	Segment-Primer/probe	Sequence (5′–3′)
UHV-1	S seg FWD	5′-ACAAACTGAATAAGACTGCTGCATT-3′
	S seg REV	5′-AGGGCTATACACACATAGTTGGATG-3′
	S seg probe	5′-6-Fam-TCCTCTGCCGCAAAAGACTATGTCACAG-BHQ-1-3′
UHV-1 &-2	L seg FWD	5′-TTGGGGAGTTTGTTACCAATGT-3′
UHV-1	L seg REV	5′-CTGAAGTCGGTCCAAATAATAAACCT-3′
	L seg probe	5′-6-Fam-TTCCCTAGGTCCACCCACTTGTTCTTTTATG-BHQ-1-3′
UHV-2	S seg FWD	5′-GCAAAACAGAACTGCTGCAGTC-3′
	S seg REV	5′-TGCGATACAGACATAATTAGAGACATTG-3′
	S seg probe	5′-6-Fam-GTCACCATGTGTCCCTCAGAACTCATTCA-BHQ-1-3′
	L seg REV	5′-GTGGGCCCAAATAACAAACCT-3′
	L seg probe	5′-6-Fam-CTCTCTCGGACCTCCCACTTGTTCCTTTATG-BHQ-1-3′
ABV-1	S seg FWD	5′-CCGTACTGCACAACTGATGATG-3′
	S seg REV	5′-AGCAACACAGGAGTAACCTGTCAC-3′
	S seg probe	5′-6-Fam-CATGAATTCTTCATCGACATCAGAAACCG-BHQ-1-3′
	L seg FWD	5′-AAAAGATCTTGCAATCCTCTTCA-3
	L seg REV	5′-GGGCCCAGAGAACTATATGT-3
	L seg probe	5′-6-Fam-TGGAGAACTTTTTGTCTGAGTTGAGGCA-BHQ-1-3′
UGV-1 &-2	S seg FWD	5′-CAAGAAAAACCACACTGCACA-3′
	S seg REV	5′-AACCTGTTGTGTTCAGTAGT-3′
UGV-1	S seg probe	5′-6-Fam-CTCGACAAGCGTGGGCGGAGG-BHQ-1-3′
UGV-2	S seg probe	5′-6-Fam-CGAGCACGGTCAAAGGGGATGAAGAG-BHQ-1-3′
UGV-1 &-2	L seg FWD	5′-TCATAAAAGCTTCTAGCTATTCTTTTCAT-3′
	L seg REV	5′-CAAGTTGGAGGCCCAAGAG-3′
UGV-1	L seg probe	5′-6-Fam-TGAAGTCTCCTCCAAGACCCTGGTTATCAG-BHQ-1-3′
UGV-2	L seg probe	5′-6-Fam-TTAGGCAACAAGCTTCATAACAGCTG-BHQ-1-3′
HISV-1	S seg FWD	5′-CTCAAAATCTTACCGAAGTTGTATGTAC-3′
	S seg REV	5′-CACTTTCCCTTTTGGATCTTTG-3′
	S seg probe	5′-6-Fam-GTGACGACCAAGTGTCGGGTCACAC-BHQ-1-3′
	L seg FWD	5′-GAGTCTTTGTTTGATAATGGTGGTT-3′
	L seg REV	5′-ATTGAAGACTACAGAACCATATC-3
	L seg probe	5′-6-Fam-TCATTTGATTCAAGTGTTCTCAGGGCA-BHQ-1-3′

### Passaging of arenavirus-infected cells generated persistently infected cell cultures as judged by the presence of viral S and L segment RNA.

To study if reptarenaviruses or hartmaniviruses could establish a persistent infection in cultured host cells, we inoculated Boa constrictor kidney cells (I/1Ki) with a virus stock containing a single reptarenavirus (UGV-1), two reptarenaviruses (ABV-1 and UHV-1), or a reptarenavirus (UHV-2) and a hartmanivirus (HISV-1). At 10 days postinfection (dpi), we collected 1/3 of the cells for RNA extraction and 1/3 to analyze protein expression and continued incubating the remaining 1/3 of cells in a new flask. After the remaining cells became confluent, we collected samples as above, placed the remaining cells in a new flask to grow until confluent, and continued the cycle for 10 consecutive cell passages. While the reptarenavirus-infected cells did not show any cytopathic effect (CPE) during the passaging of the cells, we observed cytopathic effects (cell rounding and detachment) and slowed growth in the first few passages in the cells initially inoculated with a combination of UHV-2 and HISV-1. We named the generated persistently infected cell lines PIwUHV (inoculated with UHV-1 and ABV-1), PIwUGV-1 (inoculated with UGV-1), and PIwSn11 (inoculated with HISV-1 and UHV-2).

**FIG 2:**
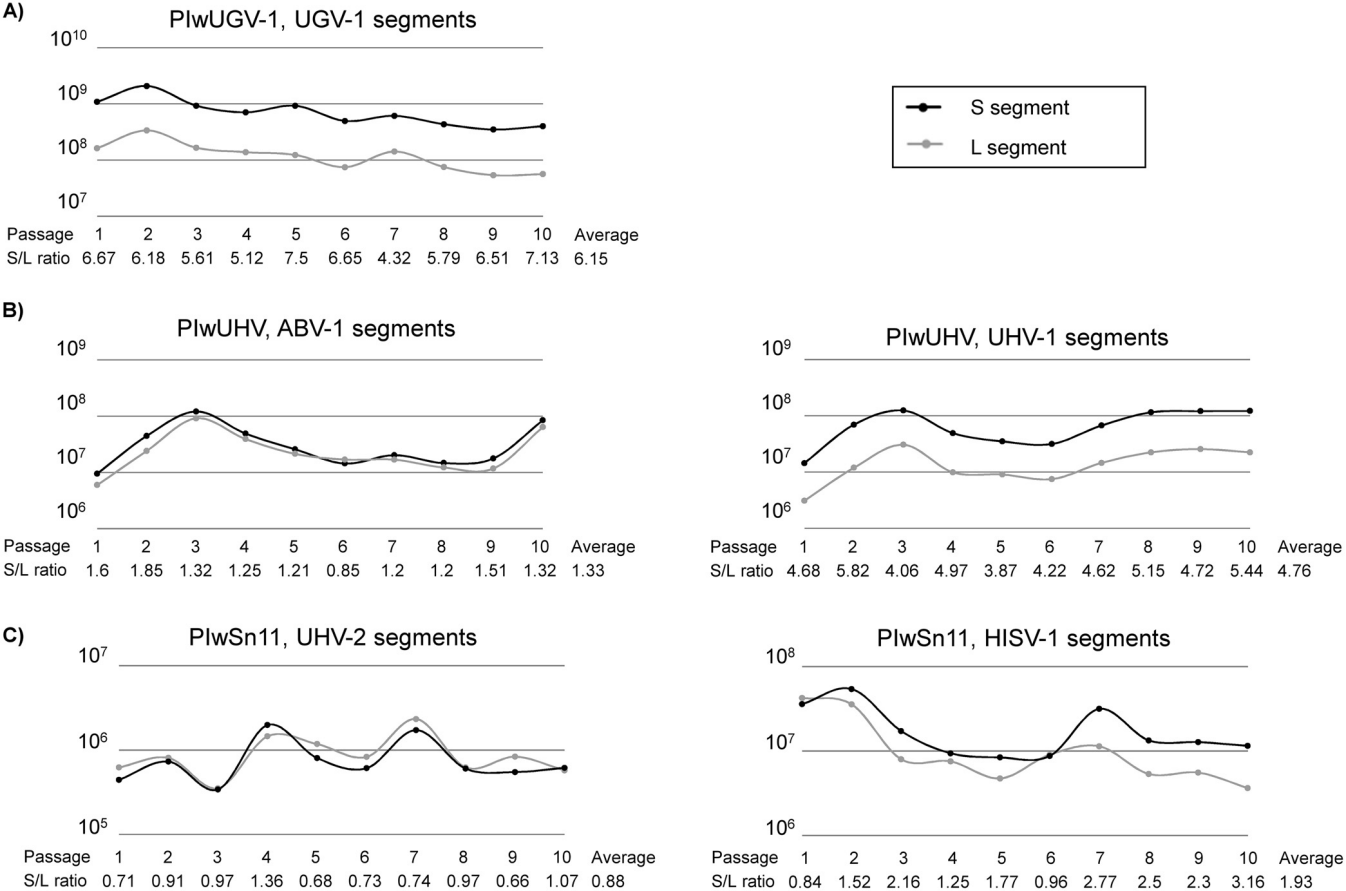
Cellular level of S and L segments through the first 10 passages of the infected cells. The RNA extracted from the infected cells at each cell passage served as the template for qRT-PCR targeting the S or L segments of the virus or viruses used in initial inoculation and the housekeeping gene GAPDH (glyceraldehyde 3-phosphate dehydrogenase). The plots show the S (black line) and L (gray line) segment copy numbers per reaction normalized against GAPDH (y-axis) at passages 1 to 10 (x-axis), and the S/L ratio below the cell passage number shows the amount of S to L segment RNA at each passage. The plots show the results of (A) single reptarenavirus infection, PIwUGV-1, qRT-PCR for UGV-1 segments, (B) dual reptarenavirus infection, PIwUHV, qRT-PCR for ABV-1 (left) and UHV-1 (right) segments, and (C) reptarenavirus-hartmanivirus coinfection, PIwSn11, qRT-PCR for UHV-2 (left) and HISV-1 (right) segments.

We employed segment-specific qRT-PCR to measure the S and L segment RNA levels with respect to a housekeeping gene (GAPDH, glyceraldehyde 3-phosphate dehydrogenase) over the first 10 cell passages. The results shown in Fig. 2 indicate that the S and L segment RNA levels remain high even at cell passage 10. The single-infected PIwUGV-1 cells (Fig. 2A) showed a steady decline in the viral RNA level after passage 2 with a passage 10 level that was approximately half of the one of passage 1. The double infected PIwUHV cells (Fig. 2B) demonstrated fluctuating S and L segment levels for both ABV-1 and UHV-1. UHV-1 segment levels appeared to stabilize at a high level from cell passage 8 onwards, whereas the ABV-1 segment levels increased in the last cell passage after a steady decline after passage 3. The PIwSn11 cells (Fig. 2C), infected with both a reptarenavirus (UHV-2) and a hartmanivirus (HISV-1) showed similar fluctuating viral RNA levels of both viruses. Like the UGV-1 RNA level in the PIwUGV-1 cells, the HISV-1 RNA level declined (~90% for the L segment and ~60% for the S segment) toward cell passage 10. However, the UHV-2 RNA level fluctuated substantially over the entire examination period, without a clear trend. Regardless of the different trends in RNA levels, the results indicate that passaging of the infected cells leads to persistent infection with the viruses tested. We also compared the ratio of S and L segments within the cells during the passaging and did not observe marked changes in the ratio of any of the pairs. While most viruses showed an average S to L segment ratio of approximately 1 to 2, the UHV-1 and UGV-1 infected cells demonstrated significantly higher ratios, 4.76 and 6.15 on average throughout cell passages 1 to 10 (Fig. 2).

### Antisera against reptarenavirus GP2 and UGV-1 GPC showed good cross-reactivity against reptarenavirus GPCs.

**FIG 3:**
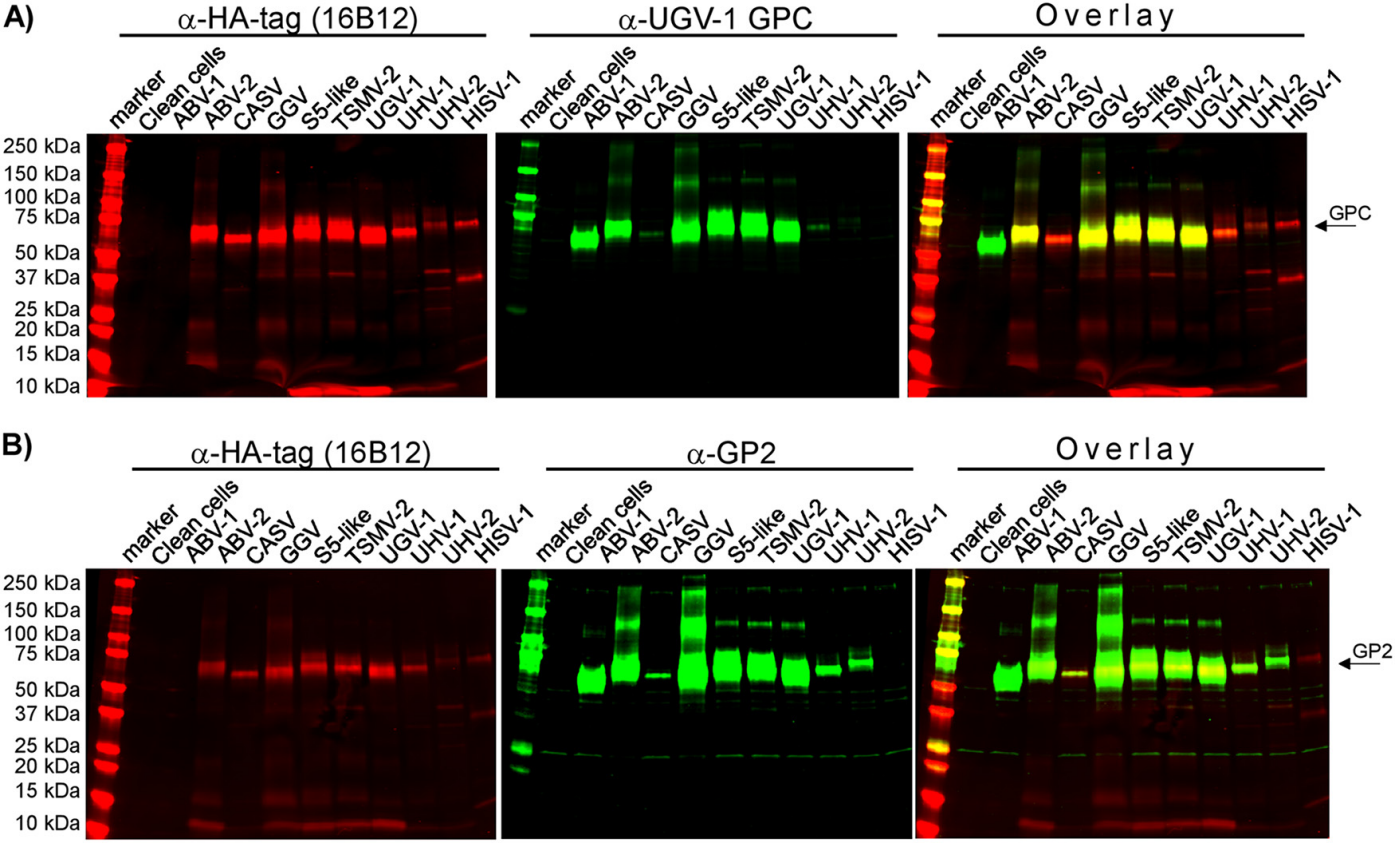
Cross-reactivity of anti-GP2 and anti-UGV-1 GPC sera against different reptarenavirus GPCs in immunoblot. Lysates of HEK293T cells transfected with plasmids encoding the GPCs of various reptarenaviruses and HISV-1, generated in an earlier study (24), were separated on precast 4% to 20% Mini-Protean TGX Precast Proteins Gels (Bio-Rad), transferred on nitrocellulose and probed simultaneously with monoclonal antibody (clone 16B12) against HA-tag and (A) anti-GP2 or (B) anti-UGV-1 GPC antiserum. The membranes were probed with IRDye 800CW Donkey anti-rabbit IgG (LI-COR Biosciences) and Alexa Fluor 680-labeled donkey anti-mouse IgG (ThermoFisher Scientific) secondary antibodies were scanned using Odyssey Infrared Imaging System (LI-COR Biosciences) to enable simultaneous detection of the mouse (the HA-tag in C terminus of the recombinant GPCs) and rabbit antibodies (the GPCs). The left shows the anti-HA tag staining in red (ABV-1 GPC is with FLAG-tag), the middle shows the staining with rabbit antiserum in green, and the right shows an overlay of the HA-tag and rabbit antiserum staining. The abbreviations are ABV-1/2 (aurora borealis virus 1/2), CASV (CAS virus), GGV (Golden Gate virus), S5-like (GPC from a virus homologous to S5 S segment described by Stenglein et al. (15)), TSMV-2 (Tavallinen Suomalainen mies virus 2), UGV-1 (University of Giessen virus), UHV-1/2 (University of Helsinki virus 1/2), HISV-1 (Haartman Institute snake virus 1, a hartmanivirus).

We have generated several rabbit antisera against the NPs of reptarena- (1, 20, 22) and hartmaniviruses (6, 23), which in the case of reptarenaviruses, but not hartmaniviruses show good cross-reactivity between NPs of different species. Somewhat surprisingly, GP2 appears to be the most conserved reptarenaviral protein because the GP2s of different reptarenavirus species (excluding the California Academy of Sciences virus [CASV]) show amino acid identities between 87 and 99% (6). We, therefore, decided to attempt to produce a broadly cross-reacting antiserum against the reptarenavirus GP2s by expressing a synthetic gene comprising the most conserved regions of GP2. In parallel, we produced an antiserum against the UGV-1 GPC produced in unprocessed form (i.e., not cleaved to GP1 and GP2) via expression in mammalian cells. To evaluate the cross-reactivity of the two rabbit antisera generated, we tested their ability to recognize the HA-tag-bearing recombinant GPCs of different reptarenaviruses and HISV-1 in immunoblots undertaken with lysates of HEK293T cells transfected in an earlier study (24). These showed that the anti-GP2 serum cross-reacts with all reptarenavirus GPCs (Fig. 3A). The immunoblot with anti-UGV-1 GPC serum showed a very similar result, indicating good cross-reactivity against the GPCs of other reptarenavirus species (Fig. 3B). This suggested a good response against the GP2 because the GP1s of reptarenaviruses show considerably higher sequence variation. Neither antiserum showed a reaction against HISV-1 GPC.

**FIG 4:**
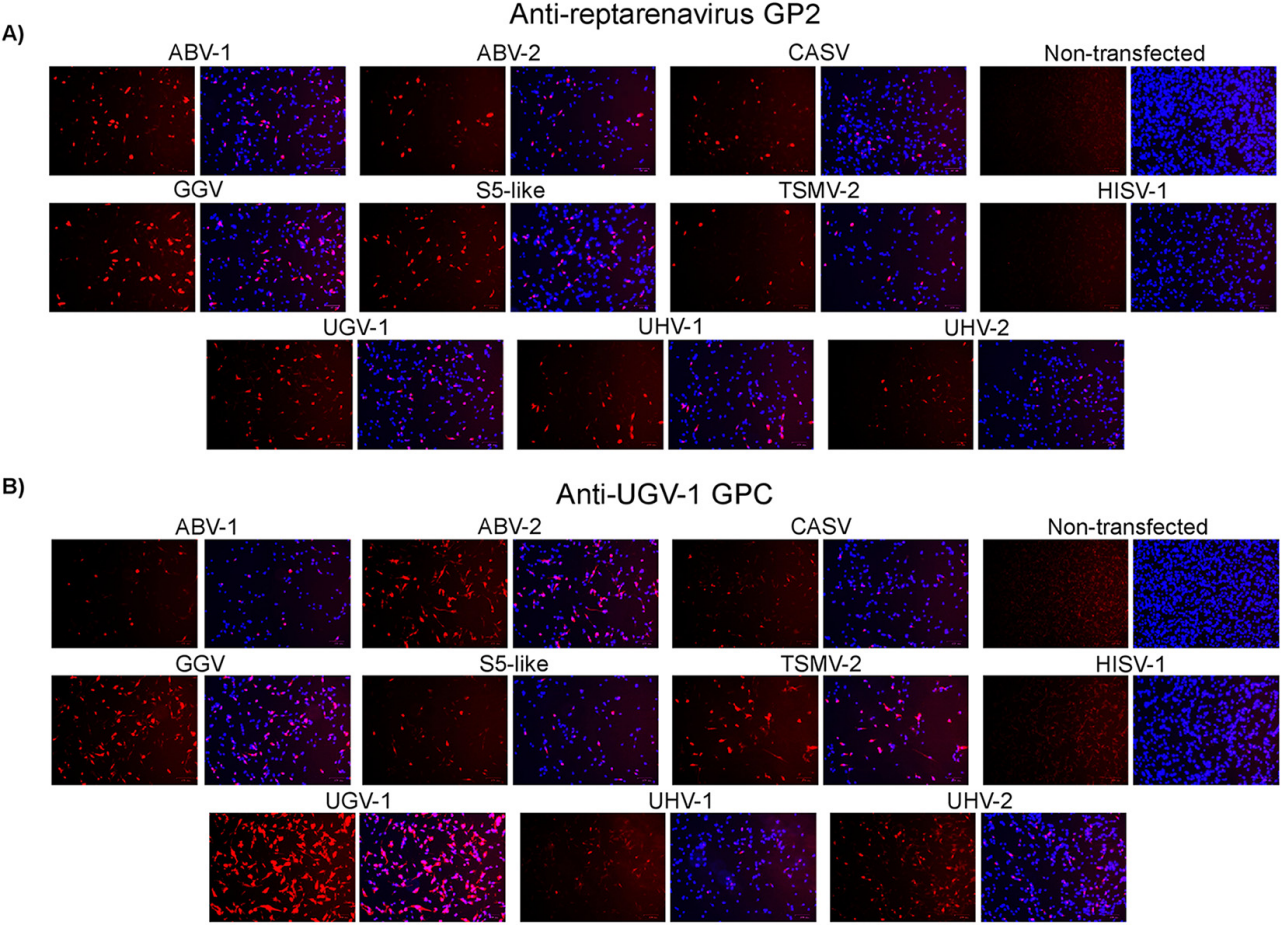
Performance of anti-GP2 and anti-UGV-1 GPC sera in immunofluorescence. I/1Ki cells transfected with plasmids encoding the GPCs of various reptarenaviruses and HISV-1, grown on collagen-coated 96-well plates, were fixed and incubated with (A) anti-GP2 or (B) anti-UGV-1 GPC antiserum. Donkey anti-rabbit IgG Alexa Fluor 594 served as the secondary antibody, Hoechst 33342 for visualizing the nuclei, and ZOE Fluorescent Cell Imager (Bio-Rad Laboratories) for recording the images. The left shows a red channel only (rabbit antiserum staining) and the right shows an overlay of the blue (nuclei of the cells) and red channel.

We next evaluated the cross-reactivity of the produced antisera in IF staining using I/1Ki cells transfected with plasmids encoding the GPCs of various reptarenaviruses and HISV-1 as the source of antigen. The results, correlated with the immunoblot finding, i.e., both antisera recognized all tested reptarenavirus GPCs but not the GPC of HISV-1 (Fig. 4A and B). In IF, at the tested dilutions, the anti-UGV-1 GPC antiserum produced less prominent staining with ABV-1, CASV, S5-like, and UHV-1 GPCs than the anti-GP2 antiserum, likely reflecting the serum’s epitope distribution.

**FIG 5:**
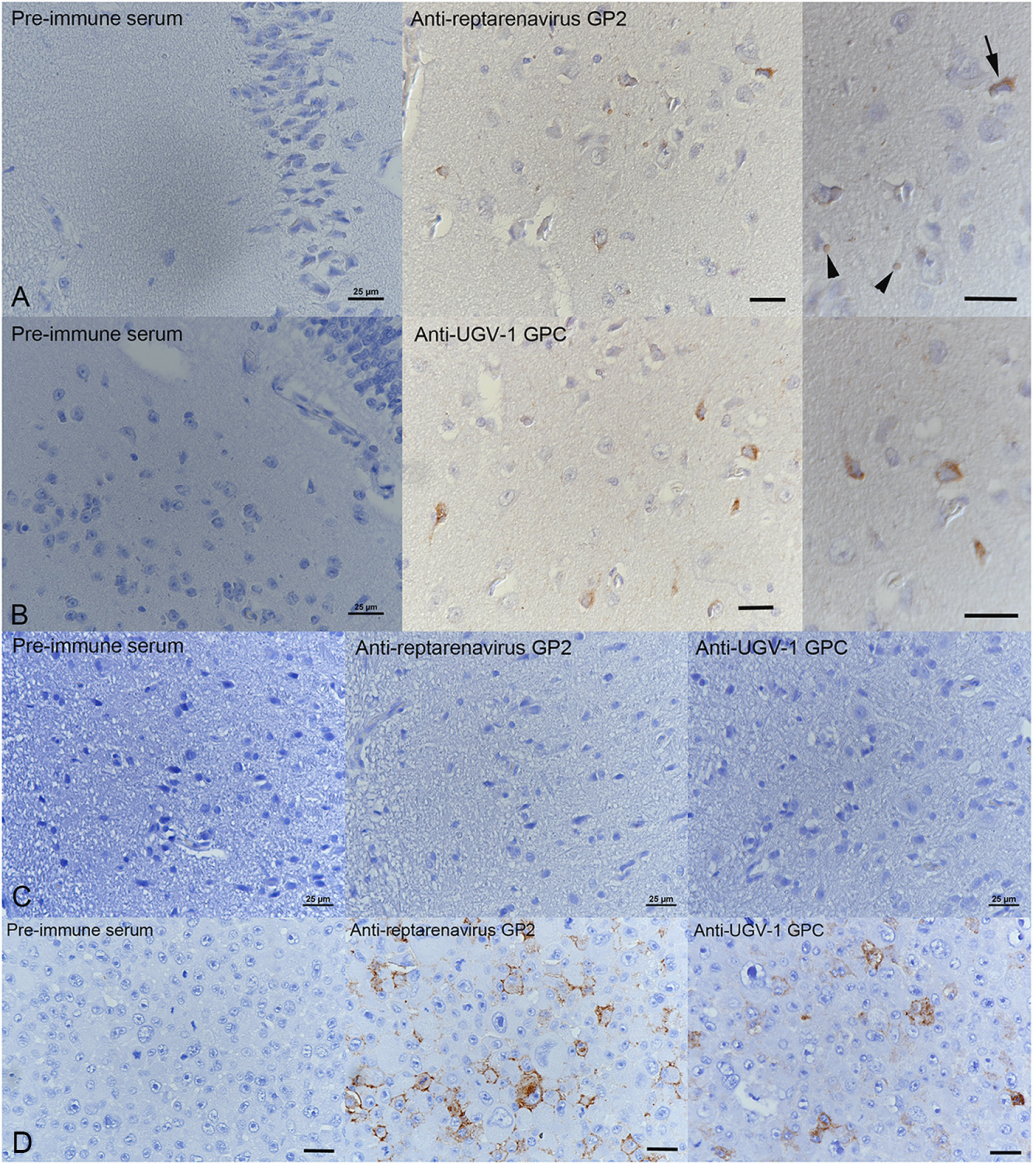
Performance of anti-GP2 and anti-UGV-1 GPC sera in immunohistochemistry. (A and B) Immunohistological staining of the brain from a boa constrictor with confirmed BIBD diagnosis. (A) Incubation with the preimmune serum (left) does not yield a reaction. Several neurons express viral GP2 (right) within the cytoplasm (arrow). GP2 was also weakly expressed in the inclusion bodies (arrowheads). (B) Similarly, no reaction is seen after incubation with the GPC preimmune serum (left), whereas staining with anti-UGV-1 GPC antiserum shows a moderate cytoplasmic reaction in individual neurons (right). (C) Staining sections of the brain from a BIBD-negative boa constrictor does not yield any reaction. (D) Both viral GP2 and GPC are expressed within boa constrictor (I/1Ki) cells infected with UGV-1 and harvested at 6 days postinfection. Immunocytochemistry, hematoxylin counterstain. Bars = 25 μm.

Finally, we tested the generated antisera in immunohistology by staining brain sections of a Boa constrictor diagnosed with and euthanized due to BIBD. Corresponding sections from a BIBD-negative boa constrictor served as controls. Staining with preimmune sera from the animals used for producing the hyperimmune sera against GP2 and GPC did not produce staining in the BIBD-positive brain section (Fig. 5A and B, left). Both anti-GP2 and anti-GPC hyperimmune sera produced a positive reaction in the neurons of a brain section from a BIBD-positive Boa constrictor (Fig. 5A and B, middle and right). Neither the preimmune nor the hyperimmune sera produced positive staining in the case of a brain section from a BIBD-negative Boa constrictor (Fig. 5C). To assess the applicability of the antisera in immunohistology further, we stained a pellet of UGV-1 infected I/1Ki cells with preimmune and hyperimmune sera. Only the hyperimmune sera produced staining in the pellet of infected cells (Fig. 5D) further supporting staining specificity.

### The expression of viral proteins during the first 10 cell passages.

**FIG 6:**
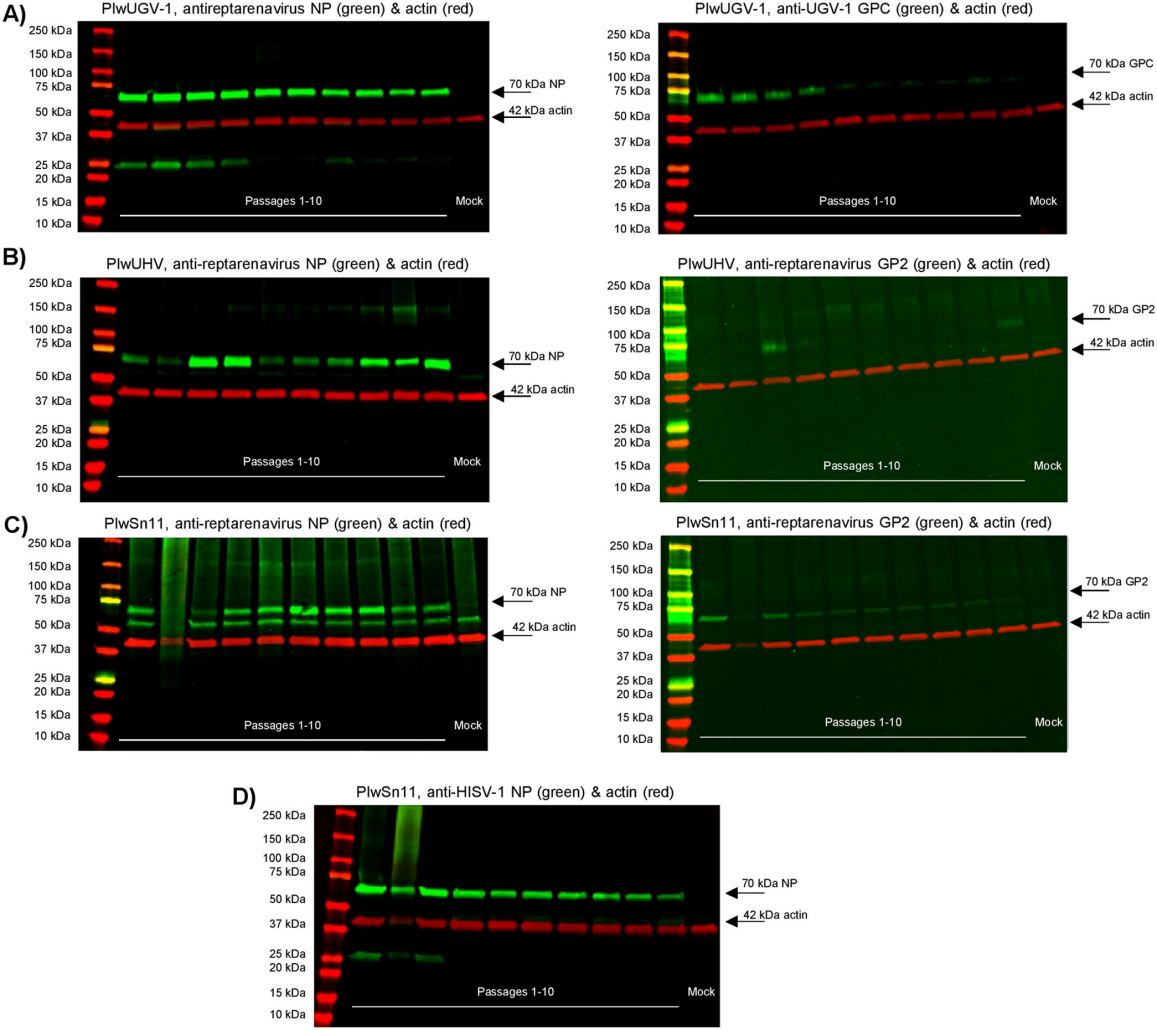
Expression of viral NP and GPs through the first 10 passages of the infected cells. Lysates of the cell pellets collected at each cell passage were separated on precast 4% to 20% Mini-Protean TGX Precast Proteins Gels (Bio-Rad), transferred on nitrocellulose and the membranes probed with rabbit anti-reptarenavirus NP, anti-HISV-1 NP, anti-UGV-1 GPC, or anti-reptarenavirus GP2 as indicated. Probing with mouse monoclonal anti-pan actin antibody served as an internal loading control. Probing with IRDye 800CW Donkey anti-rabbit IgG (LI-COR Biosciences) and Alexa Fluor 680-labeled donkey anti-mouse IgG (ThermoFisher Scientific) secondary antibodies facilitated the recording of the results with Odyssey Infrared Imaging System (LI-COR Biosciences) and enabled simultaneous detection of proteins detected with the rabbit antisera (in green) and mouse monoclonal antibody (actin in red). The plots show immunoblot of (A) PIwUGV-1 pellets over cell passages 1 to 10 with anti-reptarenavirus NP (left) and anti-UGV-1 GPC (right) in green and anti-actin in red, (B) PIwUHV (dual reptarenavirus infection with ABV-1 and UHV-1) pellets over cell passages 1 to 10 with anti-reptarenavirus NP (left) and anti-reptarenavirus GP2 (right) in green and anti-actin in red, (C) PIwSn11 (reptarenavirus-hartmanivirus coinfection) pellets over cell passages 1 to 10 with anti-reptarenavirus NP (left) and anti-reptarenavirus GP2 (right) in green and anti-actin in red, and (D) PIwSn11 pellets over cell passages 1 to 10 with anti-hartmanivirus NP in green and anti-actin in red. All plots include, on the rightmost lane, a mock-infected cell pellet as control.

To study whether the observed fluctuation and overall decrease in S segment RNA levels would translate to the expression of viral proteins, we analyzed the collected cell pellets by immunoblot and included a mock-infected cell pellet as a control. We separated the proteins of the cell pellets collected at each cell passage by SDS-PAGE and performed immunoblots using anti-reptarenavirus NP, anti-HISV-1 NP, anti-GP2, or anti-UGV-1 GPC sera together with pan-actin antibody. The immunoblot of PIwUGV-1 cells (Fig. 6A) showed the amount of GPC to decline from passage 1 onwards and to reach an overall lower level after passage 4. The amount of NP demonstrated a rather constant level during the first 10 passages, although a slight decline toward the 10th passage of the cells, similarly to the S segment RNA levels (Fig. 2A) as quantified using the primers and probe targeting the NP open reading frame (ORF), could be supported by the result. The apparent decrease in the GPC level over the passaging led us to speculate that mRNA levels could explain the observed difference between the NP and GPC. We thus designed primers and probes targeting the GPC ORF, but the qRT-PCR results with both NP and GPC ORF targeting primers produced similar results invalidating the hypothesis.

In contrast to the single reptarenavirus infected PIwUGV-1 cells, the immunoblot of PIwUHV cells infected with two reptarenaviruses showed an increase in the NP expression until 4th cell passage, after which it declined for the next two passages before starting to increase toward the 10th cell passage (Fig. 6B). The amount of NP in the PIwUHV cells mimicked the RNA levels of ABV-1 and UHV-1 S segments (Fig. 2B), and the amount of GPC appeared to mirror the NP levels at least to some extent because the intensities increased on the 3rd and 10th cell passage (Fig. 6B). The staining with anti-reptarenavirus GP2 antiserum produced faint background bands (also in the right of Fig. 6C) above the band of interest, which according to the staining of mock-infected cells (the rightmost lane) likely represent cellular proteins.

In the case of reptarenavirus (UHV-2) and hartmanivirus (HISV-1) coinfected PIwSn11 cells, the reptarenavirus NP expression showed a peak at around the 6th cell passage, after which the level of NP appeared to stabilize (Fig. 6C). The probing with anti-reptarenavirus NP antiserum produced additional staining above and a prominently stained band below the band of interest, both of which likely represent cellular proteins because they are present also on the rightmost lane representing the staining of mock-infected cells. The expression of UHV-2 GP2 (Fig. 6C) showed a decreasing trend throughout cell passaging similar to PIwUGV-1 GPC (Fig. 6A). We observed the amount of HISV-1 NP to decline toward higher cell passages (Fig. 6D), and similarly to single reptarenavirus infected PIwUGV-1 cells the slight decline appeared to correlate with the decrease in S segment RNA levels at passage 1 versus passage 10 (Fig. 2C). The PIwSn11 cells at passage 2 were in poor condition, as reflected by the aberrant migration and unclear results in the immunoblot. However, the cell growth normalized at around the 3rd and 4th cell passage. Unfortunately, we were unable to monitor HISV-1 GPC expression due to the lack of a suitable antibody or antiserum.

### The staining pattern of reptarenavirus NP in freshly and persistently infected cells.

**FIG 7:**
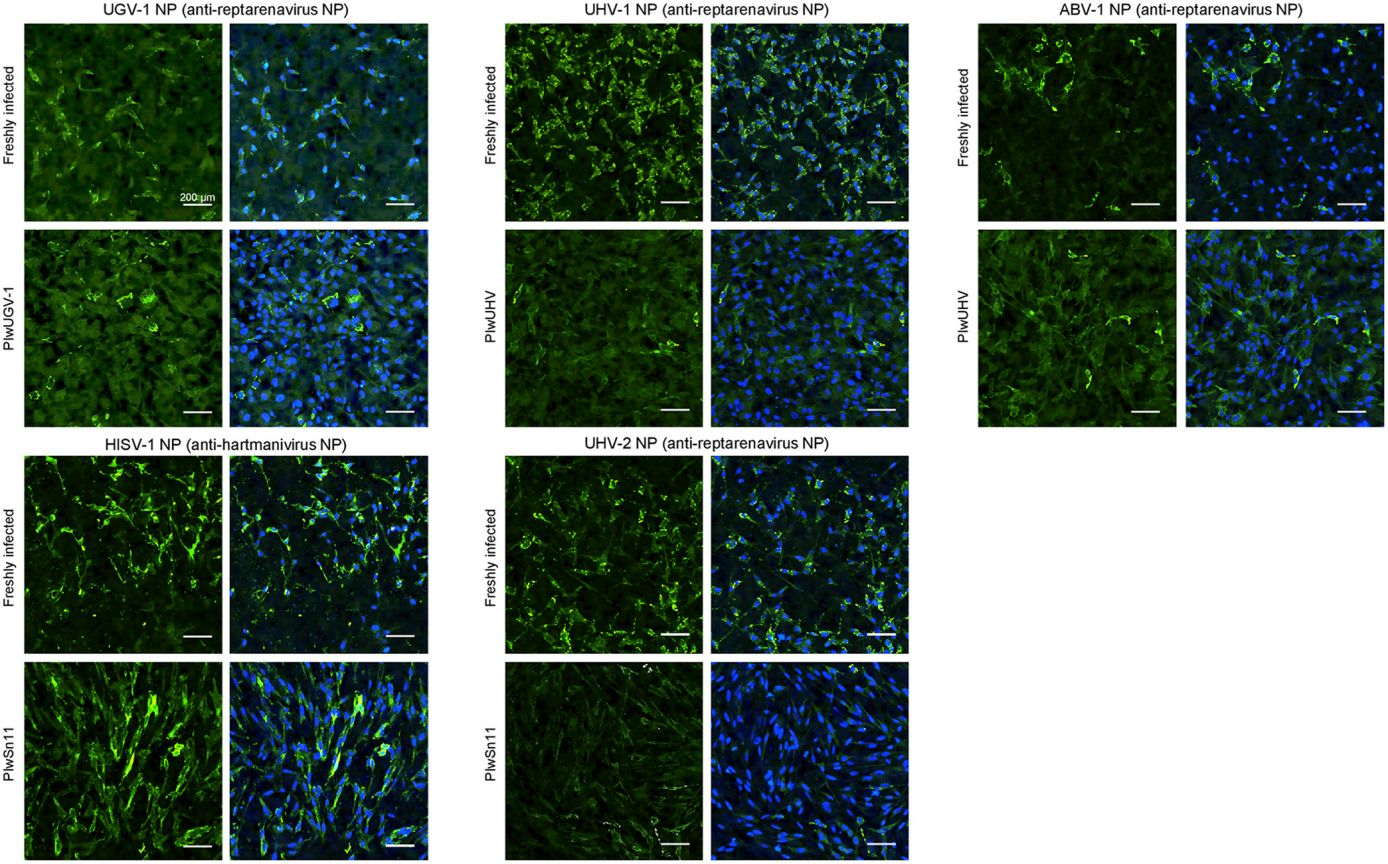
Expression of NP in the persistently infected cell lines and freshly infected I/1Ki cells. Persistently infected cells and I/1Ki cells grown on collagen-coated 96-well plates were fixed, permeabilized, and stained with rabbit antisera against reptarenavirus NP or hartmanivirus NP as indicated, Hoechst 33342 served for visualizing the nuclei. The left shows the staining against the respective NP in green, and the right shows the overlay with nuclear stain (Hoechst 33342). Opera Phenix High Content Screening System (PerkinElmer) served for capturing the images, scale bar indicates 200 μm.

Reptarenavirus infection causes BIBD (13, 14) in which cytoplasmic IBs comprising reptarenavirus NP form in various cell types of the affected animals (1, 3, 25). Studies further suggest that it may take several years from the initial reptarenavirus infection until the development of BIBD. To study the localization of NP, we compared the NP staining patterns of the persistently infected cell lines to I/1Ki cells freshly inoculated with the respective viruses by IF staining (Fig. 7). The NP staining patterns appeared similar between the freshly infected cells and persistently infected cell lines, and to our surprise, the persistently infected cell lines included a various number of individual cells with high NP expression. However, although not apparent from the present confocal images, most of the persistently infected cells appeared to stain weakly for reptarenavirus NP, as supported by the immunoblot and qRT-PCR results. Different from the granular/punctate reptarenavirus NP expression in the cells, hartmanivirus NP appeared diffusely distributed in the cytoplasm of infected cells, as observed in earlier studies (6, 23).

### The persistently infected cell lines produced infectious virions.

**FIG 8:**
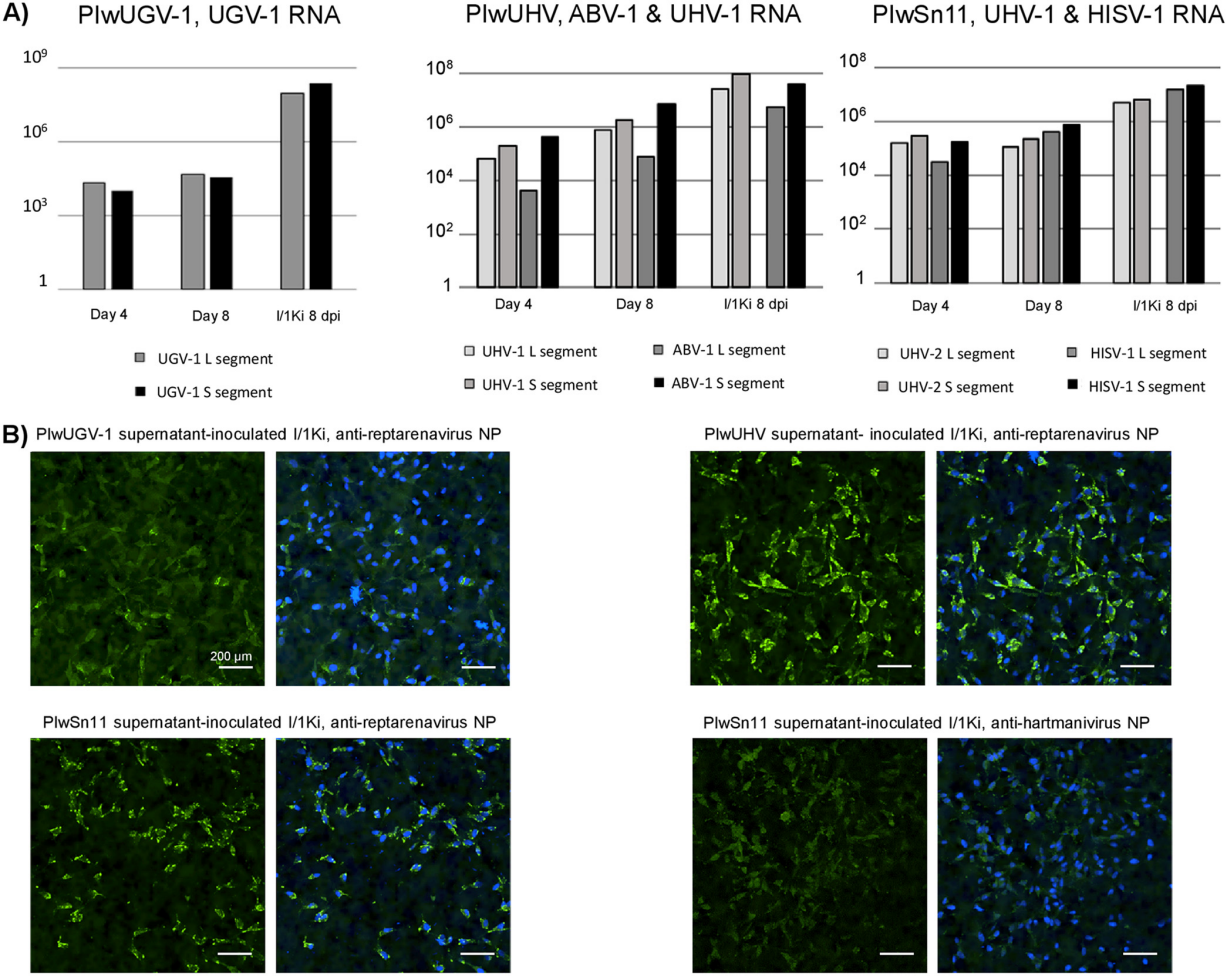
Secretion of viral RNA and infectious particles by persistently infected cells. RNA isolated from the supernatant of persistently infected cells collected at four and 8 days following medium replacement or collected 8 days postinoculation of I/1Ki cells was used as the template in qRT-PCRs targeting the S and L segments of respective viruses. (A) UGV-1 S and L segment RNA released from PIwUGV-1 cells (4 and 8 days following medium replacement) and I/1Ki cells inoculated with UGV-1 (8 dpi) (left); ABV-1 and UHV-1 S and L segment RNA released from PIwUHV cells (4 and 8 days following medium replacement) and I/1Ki cells inoculated with UHV isolate containing ABV-1 and UHV-1 (8 dpi) (middle); UHV-2 (reptarenavirus) and HISV-1 (hartmanivirus) S and L segment RNA released from PIwSn11 cells (4 and 8 days following medium replacement) and I/1Ki cells inoculated with virus isolate containing UHV-2 and HISV-1 (8 dpi) (right). (B) I/1Ki cells grown on collagen-coated 96-well plates were inoculated with supernatants of the persistently infected cells, fixed 4 days postinoculation, permeabilized, and stained with rabbit antisera against reptarenavirus NP or hartmanivirus NP as indicated, Hoechst 33342 served for visualizing the nuclei. The left shows the staining against the respective NP in green, and the right shows the overlay with nuclear stain (Hoechst 33342). Opera Phenix High Content Screening System (PerkinElmer) served for capturing the images, scale bar 200 μm.

The presence of both S and L segment RNA together with prominent NP expression in the passaged cultures indicated the establishment of persistently infected cell cultures. However, the decreased expression level of viral GPs led us to study whether the persistently infected cultures can produce infectious virions. Thus, we compared the amounts of viral RNA released into the cell culture medium from freshly and persistently infected cells at 4 and 8 days postinoculation or replacement of the growth medium in the case of the persistently infected cultures. S and L segment qRT-PCRs showed that the amount of viral RNA released from the persistently infected cells was10 to 10,000 times lower than from freshly infected cells (Fig. 8A). The UHV-2 RNA level in the supernatants of PIwSn11 cells had already reached a plateau at 4 days post-medium exchange, while the HISV-1 RNA level showed a 10-fold increase between days 4 and 8. In comparison, I/1Ki cells freshly inoculated with the respective viruses displayed 10 to 100-fold higher RNA levels at the studied time points. The supernatant collected from PIwUHV cells showed a 100-fold increase in the amount of both UHV-1 and ABV-1 RNA between days 4 and 8. However, the RNA levels of both viruses remained approximately 100 times lower than the supernatant collected on day 8 from freshly infected I/1Ki cells. The PIwUGV-1 cells demonstrated the most dramatic difference in their ability to release viral RNA compared to freshly infected I/1Ki cells, the difference being approximately 10,000-fold at 8 days post passaging and inoculation. We next tested whether the RNA secretion was associated with infectivity by inoculating I/1Ki cells with supernatants originating from the persistently infected cell lines and used IF staining for the respective NPs to demonstrate infectivity. The data in Figure 8B demonstrated that the persistently infected cell lines produce infectious particles, as expected based on the secretion of RNA.

### Cell size distribution suggested persistent hartmaniviruses but not reptarenaviruses caused alterations in cell morphology.

**FIG 9:**
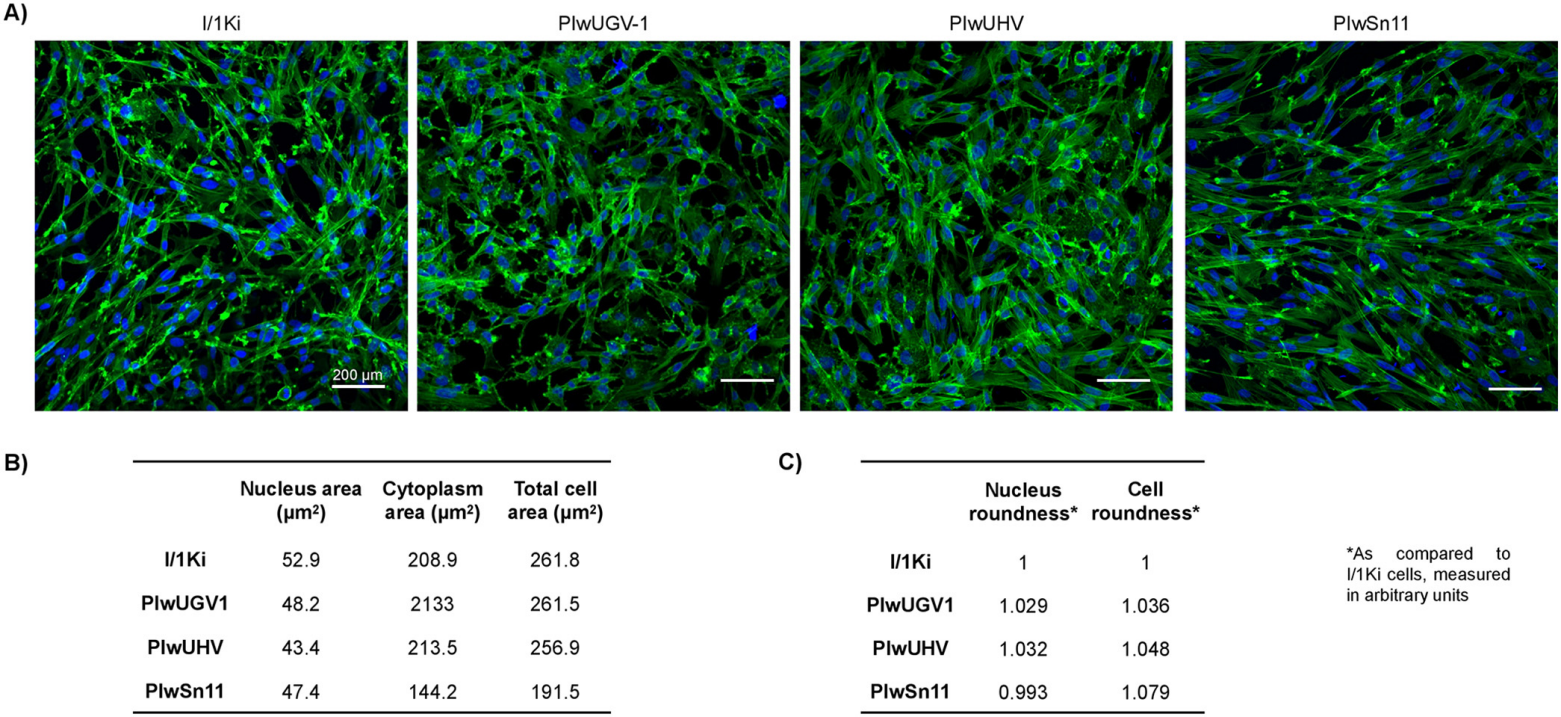
Comparison of the morphology of persistently infected cell lines to that of naive I/1Ki cells. Persistently infected and naive I/1Ki cells were grown on collagen-coated 96-well plates, fixed 2 days after plating the cells, actin cytoskeleton stained with Alexa Fluor 488 phalloidin (ThermoScientific), and nuclei visualized using Hoechst 33342. Opera Phenix High Content Screening System with (PerkinElmer) served for capturing the images (scale bar 200 μm) and for the morphological measurements of cells, cytoplasm, and nuclei. (A) An overview of the cells stained for actin cytoskeleton (in green) and the nuclei (blue). (B) Cell size distribution of the persistently infected cell lines (PIwUGV-1, PIwUHV, and PIwSn11) and naive I/1Ki cells. (C) The cell and nuclei roundness of the persistently infected cell lines (PIwUGV-1, PIwUHV, and PIwSn11) and naive I/1Ki cells.

We observed that hartmanivirus, but not reptarenavirus, infection-induced cytopathic effect (CPE) on I/1Ki cells. Thus, we studied whether the persistent infection would induce CPE or associate with alterations in the cellular size or morphology. To that end, we stained the actin skeleton of the persistently infected cell lines and uninfected I/1Ki cells and used automated high-content image analysis to determine the area and roundness of both nuclei and cytoplasm (Fig. 9). The results showed the average area of PIwUGV-1 (261.5 μm^2^) and PIwUHV cells (256.9 μm^2^) did not differ significantly from the uninfected I/1Ki cells (261.8 μm^2^) (Fig. 8). Interestingly, the hartmanivirus-reptarenavirus coinfected PIwSn11 cells (191.5 μm^2^) appeared to be on average approximately 25% smaller than all other cell lines. The cell lines did not show marked differences in the shape (roundness) of cells or nuclei.

## DISCUSSION

The identification of reptarenaviruses as the causative agent of BIBD in the early 2010s set off a series of discoveries expanding the diversity of known arenaviruses. Of the four arenavirus genera, the mammarenaviruses are by far the best characterized in terms of host tropism and molecular virology. Mammarenaviruses cause a persistent infection in their rodent hosts (21), and studies suggest that reptarenaviruses (13–16, 18, 22) and hartmaniviruses (16, 18) may act similarly. In this study, we wanted to test if reptarenaviruses and hartmaniviruses could establish a persistent infection in cell culture, as has been reported for some mammarenaviruses (26–29). We utilized cultured cells of the putative host, B. constrictor, for the study because our earlier data indicated that these cells efficiently support the replication of both reptarenaviruses and hartmaniviruses (1, 6). Passaging of cells inoculated with an isolate containing a single reptarenavirus (UGV-1), two reptarenaviruses (ABV-1 and UHV-1), or a reptarena- (UHV-2) and a hartmanivirus (HISV-1) resulted in persistent infection of the cells as judged by detection of both viral RNA and antigens over 10 cell passages. Of note, we followed the cell lines over 20 to 30 rounds of passaging and the cells remain antigen-positive and continue to secrete infectious particles and viral RNA, albeit at lower levels than freshly inoculated cells.

Animals with BIBD frequently carry multiple reptarenavirus S and L segment pairs (5, 15, 16, 18, 22). The cell culture work of Stenglein et al. (15) suggests there would be little or no constraints in the pairing of different S and L segments. Furthermore, their studies indicated that the various L segments would replicate with equal efficacy in coinfection, suggesting that they would not compete with or restrict the replication of each other (15). In this study, we demonstrated that cells coinfected with a pair of reptarenavirus S and L segments maintained both segment pairs at roughly equal levels, suggesting that the segments would not compete at the replication level. The lack of overt competition in replication between the different S or L segments fits well with the observation of frequent reptarenavirus coinfections in snakes with BIBD (5, 15), and it could represent one of the factors explaining the co-existence of multiple segments. The observation of Stenglein et al. (15) that S segments (or S6/UGV-like S segments in particular) can freely reassort with various L segments, adds another important piece to the puzzle by explaining how and why most snakes with BIBD carry more L than S segments. The S segment encodes the GPC, which following cleavage and maturation gives rise to GP1 and GP2 that form the virion’s trimeric spike complex, which in the case of mammarenaviruses (30) and likely hartmaniviruses (6) also include a stable signal peptide. Because the GPs mediate binding and entry to the host cell, one could speculate that the S segment is under higher selection pressure than the L segment, and the S segment carrying the GPC that most efficiently mediates entry would be enriched in coinfection if the segment can pair up freely. Based on the sequencing studies, the S6/UGV-like S segment is present in most (>75%) snakes with BIBD (15, 18), suggesting that the S6/UGV-like GPC efficiently mediates entry into various cell types of the Boa constrictor. However, our earlier study using recombinant vesicular stomatitis viruses pseudotyped with reptarenavirus GPs did not demonstrate significant differences in the ability to mediate entry into cells originating from various Boa constrictor tissues (17). The findings could suggest that different reptarenavirus GPCs mediate entry into various tissues equally well or that factors other than entry play a significant role in mediating tissue tropism.

In addition to GPC, the S segment encodes the NP, which plays a significant role in the replication and translation of the arenavirus genome (31–33). Thus, the NP best suited for interacting with RdRps of different reptarenavirus L segments (or reptarenavirus species) could drive enrichment of a given S segment in a coinfection setting. Our results demonstrated a slight fluctuation in S and L segment RNA levels throughout 1 to 10 cell passages of cells infected with a single reptarenavirus. Conversely, both dual reptarenavirus infection and reptarenavirus-hartmanivirus coinfection demonstrated higher fluctuation of S and L segment RNA levels, which could indicate an interplay between different S and L segments. In both cases, the S and L segments of a given reptarenavirus fluctuated similarly, which implies that the S and L segments contribute equally to replication and might show preferential pairing. The RNA level fluctuation over cell passages 1 to 10 could also relate to the spreading of the infection within the cell population or innate immune mechanisms. The NP and ZP of mammarenaviruses dampen the innate immune response by interacting with mediators of the interferon response (34). We do not know if the interferon signaling is functional in the Boa constrictor kidney cell line (I/1Ki) utilized in this study, but one could speculate that the observed fluctuation mirrors the battle between the virus and the cells’ innate immune response. We further measured the S to L segment ratio in the infected cells over passages 1 to 10 and found that different S and L segment pairs appeared to maintain a rather constant ratio. To our surprise, the S to L segment ratio showed a rather high variation between viruses, ranging from an average of 0.88 (UHV-2) to 6.15 (UGV-1) times more S than L segment RNA being present within the infected cell population. A larger amount of S segment RNA could facilitate infectious particle formation due to increased production of structural proteins of the virion, however, the higher S segment levels did not appear to translate into larger amounts of viral RNA released into the cell culture.

During the establishment of the persistently infected cultures, the S and L segment RNA levels showed an overall decreasing trend toward the 10th passage of the cells for the single reptarenavirus infection, and in the case of reptarenavirus-hartmanivirus coinfection. However, in the case of dual reptarenavirus infection, the RNA levels of both viruses appeared slightly higher at the 10th cell passage. It is possible that the dual reptarenavirus culture did not reach a steady state in terms of virus replication during the 10 rounds of cell passaging, which could explain the observed greater fluctuation in the RNA levels. Hypothetically, the fluctuation in RNA levels could have stabilized in the following passages, eventually resulting in lower S and L segment RNA levels. At the same time, it is interesting to speculate that the interplay of two reptarenaviruses or multiple reptarenavirus S and L segments would lead to higher overall RNA levels. Such a phenomenon could contribute to the accumulation of viral proteins, with the formation of NP-containing IBs as one potential consequence. The decline and fluctuation in viral RNA or S segment level did not appear to translate directly into protein expression. In the case of single and dual reptarenavirus infection, i.e., PIwUGV-1 and PIwUHV cells, the amount of NP appeared to follow the viral RNA levels to some extent. However, in the case of reptarenavirus-hartmanivirus coinfection, i.e., in PIwSn11 cells, the NP amount did not mimic the RNA level as closely. Interestingly, the number of GPs in the cells did not appear to be bound to the NP level, although the S segment encodes both proteins. In the case of PIwUGV-1, we utilized primers and probes targeting both NP and GPC ORFs of the S segment and did not find differences in the RNA levels, suggesting that the observed difference in protein level would not necessarily be related to the latter. Earlier studies have described persistently infected cell cultures for several mammarenaviruses (26–29, 35). The researchers have associated the decline in viral antigen expression in the persistent cultures with the production of defective interfering (DI) particles (28, 35) that could proposedly contribute to viral persistence in the host (36). The exact inhibition mechanism of DI particles remains unknown, but the incorporation of deletion-containing genome segments appears to be one of the contributing processes (37). Studies on lymphocytic choriomeningitis virus (LCMV) interestingly demonstrate that the late domain of ZP (38) and the phosphorylation of ZP (39) specifically contribute to DI particle formation, thus helping the virus to evade the immune response during persistence. All the persistently infected cell lines produced infectious virions, but the amount of viral RNA released from the cells was, depending on the virus, approximately 10 to 1000 times less than that secreted from freshly infected cells. We think that the decline in GPC expression would best explain the decrease in particle formation or RNA release, and it is tempting to speculate that DI particle formation would contribute to changes in protein expression. We further speculate that the differential decline in the GPC versus NP expression during persistent infection could provide a direct pathogenesis mechanism for BIBD. If the lack of GPC would hinder particle formation, the accumulation of NP and subsequent IB formation would ensue. The findings call for further studies addressing the connection between GPs and NP expression in reptarenavirus-infected snakes with or without BIBD.

BIBD appears to be a progressive disease, i.e., a reptarenavirus-infected Boa constrictor eventually will develop the disease. There are several unknowns regarding the pathogenesis of BIBD, including the details of IB formation. The established cell cultures may help to identify the associated molecular mechanisms, including the role of reptarenavirus coinfections in the development of the disease. The cultures will allow studies on the replication dynamics between different reptarenavirus species and the potential role of DI particles in reptarenavirus infection. An aspect of particular interest is whether reptarenaviruses would be able to prevent replication of closely related viruses, a feature described for persistently infected mammarenavirus cultures (26, 40).

## MATERIALS AND METHODS

### Viruses and generation of persistently infected cell lines.

University of Helsinki virus 1 (UHV-1) was originally isolated from a bone marrow cell line generated from a boa (Boa constrictor) euthanized due to BIBD (1). The virus preparation used in this study was later demonstrated to contain two reptarenaviruses: UHV-1 (GenBank accession no. L segment KR870020 and S segment KR870011) and aurora borealis virus-1 (ABV-1, L segment KR870021, and S segment KR870010) at an approximately 1:1 ratio according to next-generation sequencing reads (5). University of Giessen virus 1 (UGV-1) was initially isolated from a liver sample of a BIBD-positive Boa constrictor (1) and sequenced (L segment KR870022 and S segment KR870012) in a subsequent study (5). UHV-2 (L segment KR870030 and S segment KR870016) was also isolated from a liver sample of a BIBD-positive Boa constrictor (1). A subsequent sequencing study revealed the isolate to also contain Haartman Institute snake virus-1 (HISV-1) (5) which was later sequenced completely (L segment NC_043444 and S segment NC_043443) (6) and classified as genus *Hartmanivirus* within the family *Arenaviridae* (7).

The Boa constrictor kidney cell line, I/1Ki, described in (1) was maintained in minimal essential medium Eagle (MEM, Sigma-Aldrich) supplemented with 10% fetal bovine serum (Gibco), 2 mM l-glutamine (Sigma-Aldrich), 100 IU/mL penicillin (Sigma-Aldrich), and 100 μg/mL streptomycin (Sigma-Aldrich) at 30°C and 5% CO_2_ as described (1, 5, 20). The initial inoculations with UHV (UHV-1 and ABV-1, (1, 5)), UGV-1 (1, 5), and Sn11 isolate (HISV-1 and UHV-2,) at a multiplicity of infection (MOI, measured as fluorescent focus-forming units/cell) of approximately 0.1 to 1 were done on approximately 75% to 80% confluent monolayers of I/1Ki cells grown on 75 cm^2^ flasks by adsorbing the respective virus preparations for 1 h at 30°C and 5% CO_2,_ followed by two washes with the conditioned medium, addition of fresh medium, and incubation at 30°C and 5% CO_2_ for 10 days. After incubation, the cell pellets were suspended into a fully supplemented growth medium and the cell suspension was divided into three portions, 1/3 of the cells were placed into a clean 75 cm^2^ flask for the next passage, and 1/3 of the cells were collected for immunoblot, and 1/3 of cells for RNA extraction. The cell suspensions for the next passage were diluted with fresh supplemented growth medium and incubated at 30°C and 5% CO_2_ until the monolayers became confluent (5 to 14 days depending on the passage and infected cell line). The cell samples collected for immunoblot and RNA extraction were washed three times with phosphate-buffered saline (PBS) (3 min, 500 × *g* centrifugation between washes) and stored at −80°C until lysis and RNA extraction. Passaging and sample collection were continued as described above until passage 10. The generated persistently infected (PI) cell lines were designated PIwUHV (UHV-1 and ABV-1 infected), PIwUGV-1 (UGV-1 infected), and PIwSn11 (HISV-1 and UHV-2 infected).

### Production of antisera against recombinant reptarenavirus GP2 and UGV-1 GPC.

To design an antigen for producing a broadly cross-reactive antiserum against GP2s of different reptarenaviruses, we aligned the GPCs of different reptarenavirus species and selected the two regions with the highest sequence homology. The first region SKVDNTLEPGCDSNVGLFGHSTGTD maps to approximately amino acid residues 250 to 274 of the GPC and the second region SQLEHVTDAIACKIAKTSNYTTTALFLLNKEEGEIRDHVVEHEVALNYLLAHQGGLCNVVKGPMCCSDIDDFRRNVSDMIDKVHEEMKKFYHEPD to approximately residues 295 to 389. For producing a recombinant protein in Escherichia coli, we used a codon-optimized synthetic gene encoding the above-mentioned amino acid stretches separated by five glycine residues and followed by three glycine residues in the pET-20b(+) vector from Gene Universal. The protein production and purification under denaturing conditions were done as described (41).

For producing the UGV-1 GPC through mammalian expression, we used a Gene Universal synthetic gene encoding the GPC open reading frame of UGV-1 with the transmembrane helix replaced by a T4 fibritin trimerization domain. We subcloned the insert into the pCAGGS/MCS-Zeo-fwd vector, produced a plasmid maxiprep, transfected HEK293T cells with the construct, performed Zeocin selection, adapted the cells for suspension culture, and produced and purified the recombinant protein as described (42).

We sent 0.5 mg of both purified recombinant antigens to BioGenes GmbH (BioGenes adheres to EU and global animal welfare regulations) for immunizing one rabbit with one antigen according to the immunization scheme applied earlier (6, 41).

### Cell lysis and immunoblot.

The cell pellets stored at −80°C were lysed in Tris-buffered saline (50 mM Tris, 150 mM NaCl, pH 8.0) with 1% Triton X-100 and EDTA-free Protease Inhibitor Cocktail (Roche). The protein concentrations were measured using the Pierce BCA Protein assay kit (ThermoFisher Scientific) according to the manufacturer’s protocol. The samples were mixed with Laemmli sample buffer, and the proteins (10 μg of total protein per lane) were separated on 4% to 20% Mini-Protean TGX Precast Proteins Gels (Bio-Rad) under standard conditions. Rabbit anti-pan reptarenavirus antiserum (1:2000 dilution), cross-reacting well with different reptarenavirus NPs, described in (43), and rabbit anti-HISV-1 NP antiserum (1:2000 dilution) were used for the detection of reptarenavirus and hartmanivirus NP in immunoblots. Rabbit anti-reptarenavirus GP2 and anti-UGV-1 GPC served to detect the reptarenavirus GPs. For loading control, we employed Lab Vision pan-actin mouse monoclonal antibody (42 kDa) (ThermoFisher Scientific) at 1:200 dilution as described in (43). The secondary antibodies employed were IRDye 800CW donkey anti-rabbit (IgG) (LI-COR Biosciences) and Alexa Fluor 680-labeled donkey anti-mouse (IgG) (ThermoFisher Scientific), both used at 1:10000 dilution. SDS-PAGE, immunoblotting, and recording of results were performed as described (17, 24, 43). For testing the generated anti-reptarenavirus GP2 and anti-UGV-1 GPC sera, we employed the lysates of HEK293T cells transfected with various reptarenavirus GPC (24) that had been stored in Laemmli sample buffer at −20°C for approximately 2 years.

### Generation of control RNA for quantitative reverse transcription PCR (qRT-PCR).

We ordered the following synthetic genes under the T7 promoter (TAATACGACTCACTATAG) followed by the PmeI (GTTTAAAC) restriction site in the pUC57 vector from Genscript: ABV-1 S segment (468 nucleotides, 3 to 350 of GenBank accession no. KR870010), ABV-1 L segment (455 nt, 3263 to 3717 of KR870021), UHV-1 S segment (453 nt,457 to 909 of KR870011), UHV-1 L segment (459 nt, 3297 to 3755 of KR870020), UHV-2 S segment (468 nt, 495 to 962 of KR870016), UHV-2 L segment (455 nt, 3282 to 3736 of KR870030), UGV-1 S segment (453 nt,567 to 1019 of NC_039005), UGV-1 L segment (482 nt,3214 to 3695 of NC_039006), UGV-2 S segment (437 nt,546 to 982 of KR870015), UGV-2 L segment (473 nt, 3251 to 3723 of KR870029), HISV-1 S segment (450 nt, 486 to 935 of NC_043444), and HISV-1 L segment (467 nt, 3213 to 3679 of NC_043443). For amplification, the plasmids were transformed into Escherichia coli DH5α, and after plating onto Luria Broth plates with 100 μg/mL ampicillin, single colonies were picked to produce plasmid stocks utilizing ZymoPURE II Plasmid Maxiprep kit (Zymo Research). For *in vitro* transcription, the plasmids were opened using FastDigest MssI (PmeI, ThermoFisher Scientific), and the opened plasmid was purified by agarose gel electrophoresis and the GeneJET Gel Extraction kit (ThermoFisher Scientific). The preparations were repurified using Agencourt Ampure XP beads (Beckman Coulter) and eluted into milli-Q water (Merck-Millipore) before *in vitro* transcription. The control RNAs were produced using the TranscriptAid T7 High Yield Transcription kit and purified with the GeneJET RNA purification kit, both from ThermoFisher Scientific. The RNA concentrations were determined using NanoDrop 2000 (ThermoFisher Scientific) and the conversion to copy numbers was done using an online calculation tool (available at: http://endmemo.com/bio/dnacopynum.php). The RNA stocks were stored at −80°C until use. All kits and products were used according to the manufacturer’s protocol.

### Nucleic acid extraction, primers, probes, and qRT-PCR.

For nucleic acid extraction, the cell pellets stored at −80°C were resuspended in TRIzol (ThermoFisher Scientific) and RNA and DNA were purified following the manufacturer’s protocol. The primers and probes utilized were ordered from Metabion GmbH and are listed in Table 1. To confirm that the presence of RNA from closely related reptarenaviruses does not affect the performance of qRT-PCRs with different L and S segment primer and probe combinations, we compared the Ct values of the respective control RNAs obtained in the presence and absence of the other control RNAs. The composition of qRT-PCR mixtures for samples was half of the amount from the manufacturer’s protocol (2.5 μL of TaqMan Fast Virus 1-Step Master Mix [ThermoFisher Scientific], 2.5 μL of the extracted RNA template, 4.375 μL of sterile milli-Q water [Merck-Millipore], 0.5 μM forward and reverse primers [Metabion sequences in Table 1], 0.25 μM probe). When analyzing the ability of primer and probe combinations to amplify the correct target in the presence and absence of the other control RNAs, we added carrier RNA to the reaction mixtures to better mimic the composition of the isolated RNA samples. The qRT-PCRs were run in duplicate on 4titude skirted 96-well plates (Brooks Life Sciences) with the AriaMx real-time PCR system (Agilent) (1: 5 min at 50°C; 2: 20 sec at 95°C; 3: 3 sec at 95°C; 4: 30 sec at 60°C [steps 3 and 4 were repeated 40 times]).

### Immunofluorescence (IF) staining.

For IF staining, Tissue Culture Treated ViewPlate-96 Black (PerkinElmer) or CellCarrier-96 Ultra (PerkinElmer) plates were collagen-coated by adding 50 μL/well of Collagen Type I from rat tail (BD Biosciences) at 0.1 mg/mL concentration in 25 mM acetic acid followed by overnight incubation at 4°C as described (43). After incubation, the coating solution was replaced by sterile PBS and the plates were stored at 4°C until use, when used directly, the wells were washed once with PBS before the addition of cells. I/1Ki cells transfected with pCAGGS-based constructs for producing recombinant reptarenavirus GPCs with HA-tag as described (24, 43) served to test the anti-reptarenavirus GP2 and anti-UGV-1 GPC rabbit antisera. To test the ability of the persistently infected cell cultures to produce infectious virus, we used supernatants collected 7 days post passaging of the persistent cell cultures (at or close to passage 30, depending on the cell line) to inoculate I/1Ki cells plated on 96-well plates. The cells were fixed and analyzed at 2 or 4 dpi. When analyzing the persistently infected cultures, the cells were allowed to attach in a fully supplemented growth medium at 30°C and 5% CO_2_ for at least 24 h before fixing and staining. Fixation and IF staining followed described protocols (17, 43). Rabbit anti-UHV NP-C antiserum (1:2000 dilution) described in (20), rabbit anti-HISV-1 NP antiserum (1:2000 dilution) described in (6), rabbit anti-reptarenavirus GP2 (1:500), rabbit anti-UGV-1 GPC (1:1000), and Alexa Fluor 488 labeled rabbit anti-reptarenavirus NP (1:500 dilution) described in (22) were used as primary antibodies. The secondary antibodies, Alexa Fluor 488- or 594-labeled donkey anti-rabbit immunoglobulin (ThermoFisher Scientific), were used at 1:1000 dilution. When analyzing cell morphology, Alexa Fluor 488 or Alexa Fluor 594 Phalloidin (ThermoScientific) served to stain the actin cytoskeleton following the manufacturer’s guidelines. The imaging was done using either ZOE Fluorescent Cell Imager (Bio-Rad) or the Opera Phenix High Content Screening System (PerkinElmer), a method provided by FIMM (Institute for Molecular Medicine Finland) High Content Imaging and Analysis (FIMM-HCA).

### Immunohistology with anti-reptarenavirus GP2 and anti-UGV-1 GPC antisera.

Archival paraffin-embedded tissue from a Boa constrictor with confirmed BIBD and one for which BIBD and reptarenavirus infection had been negative for viral NP by histology and immunohistology and had undergone a full diagnostic postmortem examination at the Institute of Veterinary Pathology, Vetsuisse Faculty, the University of Zürich (at the owners’ request) was used. These diagnostic motivated necropsies did not require ethical permission. Tissue specimens from the brain had been fixed in 10% buffered formalin, trimmed, and routinely paraffin wax embedded. Consecutive sections (3 to 5 μm) were prepared and stained with anti-reptarenavirus GP2 (1:8000) and anti-UGV-1 GPC (1:8000), respectively, using the staining protocols previously described (1). Sections stained with the respective preimmune serum served as negative controls. Sections from a pellet of I/1Ki cells infected with UGV-1 and harvested at day 6 postinfection, fixed in 10% buffered formalin, and routinely paraffin wax embedded were stained following the same protocols and served as positive controls.
